# Exploring depression in people with schizophrenia spectrum disorders: a cross-sectional analysis of the clinical relationship with Positive and Negative Syndrome Scale dimensions

**DOI:** 10.47626/1516-4446-2023-3418

**Published:** 2024-03-25

**Authors:** Francesco Bartoli, Angela Calabrese, Federico Moretti, Marta Castiglioni, Luca Prestifilippo, Aldo De Pietra, Marco Gazzola, Paolo Camera, Cristina Crocamo, Giuseppe Carrà, NOMIAC Investigators

**Affiliations:** 1 Department of Medicine and Surgery, University of Milano-Bicocca, Monza, Italy; 2 Department of Mental Health and Addiction Services, ASST Nord Milano, Sesto SG Hospital, Milano, Italy; 3 Division of Psychiatry, University College London, London, UK

**Keywords:** Schizophrenia, depression, psychopathology, hallucinations, delusions

## Abstract

**Objective:**

Evidence on the relationship between depression and clinical dimensions of schizophrenia remains limited. This cross-sectional study investigated the association between depression and Positive and Negative Syndrome Scale (PANSS) dimensions in people with schizophrenia spectrum disorders.

**Methods:**

Trained assessors administered the PANSS to measure symptoms of schizophrenia and the Calgary Depression Scale for Schizophrenia to measure depression. The association of depression with overall PANSS score and related dimensions was investigated in multiple logistic regression analyses.

**Results:**

We included 231 inpatients with schizophrenia spectrum disorders (mean age: 42.4 (SD: 12.9) years; men: 58.9%; mean overall PANSS score: 82.5 (SD: 20.1); drug-free or naïve: 39.3%), including 78 (33.8%) with clinically significant depressive symptoms. Depression was associated with higher overall (regression coefficient, SE: 0.029, 0.008; p < 0.001) and general psychopathology (regression coefficient, SE: 0.118, 0.023; p < 0.001) PANSS scores. We found an inverse relationship between depression and positive symptoms (regression coefficient, SE: -0.088, 0.028; p = 0.002). No association between depression and negative symptoms was found.

**Conclusion:**

Despite some limitations, our study shows that people affected by schizophrenia spectrum disorders with depression are likely to show more overall and general psychopathology symptoms but lower positive symptoms. Additional studies are needed to explore the generalizability of our findings.

## Introduction

Over the years, the traditional view of schizophrenia has considered positive and negative symptoms as separate clinical domains, identifying individuals with prominent delusions and hallucinations and those with diminished expression and anhedonia/asociality.^1^-^4^ Nevertheless, research investigating depression as an additional clinical dimension in schizophrenia spectrum disorders (SSD) have made the interplay between positive and negative symptoms clearer,^5^-^9^ challenging the traditional distinction between nonaffective and affective psychoses.^10^

Indeed, depressive symptoms are very common in people with schizophrenia: it has been estimated that around one-third of affected individuals might suffer from comorbid depression.^11^ Different interpretations of this vulnerability to depressive symptoms suggest several possible pathways, including depression as an intrinsic domain of psychosis, depression as a psychological reaction to psychosis, and depression as a result of social and psychological factors preceding psychotic onset.^12^,^13^ From a different perspective, it has been hypothesized that depression in schizophrenia might be at least partially explained by the genetic overlap between these disorders.^11^ Moreover, functional gene polymorphisms, especially those implicated in serotonergic neurotransmission, may play a role in the occurrence of depressive symptoms in people with schizophrenia.^14^ Additional claims suggest the role of high levels of mood instability and emotional reactivity in the context of maladaptive cognitive emotion regulation strategies, involving situation selection, rumination, worry, re-appraisal, and experiential avoidance.^10^

Based on current diagnostic criteria, depression is characterized by symptoms such as anhedonia, motor retardation, and reduced ability to think or concentrate,^15^ which may show some clinical overlap with negative symptoms. Nevertheless, depressive features can be differentiated from negative symptoms in schizophrenia when phenomenology is carefully considered.^16^ Although anhedonia, anergia, and avolition may be common to both depressive and negative symptoms, other core depressive symptoms are much more likely to be independent.^10^,^16^ However, current evidence about the relationship between depressive symptoms and key clinical dimensions of schizophrenia remains inconclusive.^6^,^8^,^16^,^17^ In particular, recent studies have tested depression in the context of specific subsamples of people with schizophrenia, such as those with predominant negative symptoms,^18^ with limited generalizability. Regular and systematic screening for depressive symptoms in people with schizophrenia appears essential, considering that appropriate management of mood symptoms is needed to improve disease outcomes and psychosocial functioning.^10^,^19^

To expand knowledge in this research field, we conducted a cross-sectional study investigating the association between depression, positive and negative symptoms, and general psychopathology in people with SSD.

## Methods

### Study design

This cross-sectional study followed Strengthening the Reporting of Observational studies in Epidemiology checklist.^20^

### Population and eligibility criteria

We included individuals aged 18-65 years with SSD who were consecutively admitted to either of the two psychiatric intensive care units (27 total beds) of the local ASST Nord Milano Mental Health Care Trust between May 2020 and March 2023. This service provides mental health care for about 280,000 residents of highly urbanized districts, both deprived and affluent, in the northern area of metropolitan Milan, Italy. If study participants had multiple admissions during the study period, data from the first recorded hospitalization with depression, if any, were used. We excluded individuals who were unable to understand or communicate in Italian and those affected by cognitive deficit or intellectual disability.

### Data collection

We collected information on sociodemographic characteristics, including age, sex, education, and marital and employment status. Data on clinical features, including involuntary admissions, co-occurring personality disorders, history of suicide attempts, alcohol and substance use disorders, physical comorbidities, and psychopharmacological treatments at admission were retrieved from clinical interviews, individual electronic records, and chart review. Trained assessors (belonging to the “NOMIAC Investigators” staff) identified the participants with SSD using the Structured Clinical Interview for DSM-5.^21^,^22^ Symptom severity was assessed using the Positive and Negative Syndrome Scale (PANSS), including seven items in the Positive Scale, seven items in the Negative Scale, and 16 items in the General Psychopathology Scale.^23^ For negative symptoms, we also considered alternative scoring that excludes N5 (difficulty in abstract thinking) and N7 (stereotyped thinking) items.^24^ Depressive symptoms were measured using the Calgary Depression Scale for Schizophrenia (CDSS), adopting the recommended cut-off of > 6 to diagnose clinically significant depression.^25^ Anonymized data were included in a standardized extraction template and were double-checked to ensure precision.

### Data analysis

Standard statistics, including proportions (%), means (SD) or medians (IQR) were used for descriptive purposes. Univariate analyses explored potential differences between study participants with and without a CDSS score > 6. Chi-square or Fisher's exact tests, according to the number of events, were used to compare categorical variables, while either Student's *t*-test or the Mann-Whitney *U* test, depending on data distribution, were employed for continuous variables. Multiple logistic regression models were used to estimate the association between depression (i.e., CDSS score > 6) with PANSS overall, Positive, Negative, and General Psychopathology scores, all adjusted for age, sex, and any variables with p < 0.05 at the univariate level. Additional, more restrictive, analyses were carried out for Negative score by excluding items N5 and N7. Data were checked for multicollinearity with the Belsley-Kuh-Welsch technique. Heteroscedasticity and residual normality were measured using the White and the Shapiro-Wilk tests, respectively. Statistical significance was set at p < 0.05. All analyses were performed in Stata 17.^26^

### Ethics statements

This study was approved by the local ethics committee (number 672-17112020), as a part of the broader Northern Milan Area Cohort (NOMIAC) project.^27^,^28^

## Results

### Characteristics of the study participants

A total of 258 individuals with SSD had at least one hospital admission during the study period. Of these, 3 who were under 18 years of age and 24 who were over 65 years of age were excluded, thus 231 adults with SSD were included. The mean participant age was 42.4 (SD: 12.9) years. The majority of the participants were men (n = 136; 58.9%). The CDSS score of about one-third of the sample (n = 78; 33.8%) was > 6. The mean overall PANSS score was 82.5 (SD: 20.1), with Positive, Negative, and General Psychopathology sub-scores of 21.6 (SD: 8.4), 19.1 (SD: 9.0) and 41.8 (SD: 11.3), respectively. Ninety participants (40%) were untreated (drug-free or drug-naïve) at the time of admission. The main characteristics of the overall sample are reported in Table 1.

### Correlates of depression: univariate analyses

Univariate analyses showed that a CDSS score > 6 was associated with a higher overall PANSS score (p < 0.001) and Negative (p = 0.006) and General Psychopathology sub-scores (p < 0.001). Moreover, study participants with depression were more frequently medicated with antidepressants (p < 0.001) and had higher lifetime suicide attempt rates (p = 0.017) and alcohol use disorder (p = 0.034). None of the other variables showed associations with a CDSS score > 6. The details are reported in Table 1.

### Association between depression and PANSS scores: multiple logistic regression analyses

Table 2 shows analyses adjusted for age, sex, and variables with p < 0.05 at the univariate level (antidepressant treatment, history of suicide attempts, and alcohol use disorder). A CDSS score > 6 was associated with higher overall PANSS score (regression coefficient [coeff.], SE: 0.029, 0.008; p < 0.001) and General Psychopathology scores (coeff., SE: 0.118, 0.023; p < 0.001). We also found that individuals with higher positive symptoms levels were significantly less likely (coeff., SE: -0.088, 0.028; p = 0.002) to report depressive symptoms. We found no association between depressive and negative symptoms (coeff., SE: -0.026, 0.021; p = 0.21). These results were also confirmed after excluding N5 and N7 (coeff., SE: -0.034, 0.026; p = 0.20).

## Discussion

In this cross-sectional study, based on rigorous sampling procedures and standardized assessments, we explored the association between depressive and psychotic symptoms among inpatients with SSD.

First, we found that people with clinically significant depression had higher overall PANSS symptom severity and General Psychopathology sub-scores, regardless of age, sex, or other key clinical variables, including antidepressant treatment, history of suicide attempts, and alcohol use disorder. This is not surprising and appears to be consistent with most recent evidence,^18^,^29^ showing that depressive symptoms in schizophrenia may be associated with more persistent psychotic symptoms,^30^ possibly leading to higher overall PANSS scores. Individuals with depressive symptoms may experience more difficulties in daily activities, as well as in social and occupational functioning,^29^ which may negatively influence key psychotic symptoms. Moreover, depression is likely to lead to hopelessness, social withdrawal, and negative self-perception, worsening the distressing experiences associated with psychosis.^31^ Nevertheless, the relationship between depressive and psychotic symptoms is complex and multidirectional: the psychosocial consequences of schizophrenia, such as social isolation, stigma, and impaired functioning, can contribute to the development of depressive symptoms.^10^ In addition, at least a partial overlap between depressive and psychotic domains as assessed by CDSS and PANSS General Psychopathology items, should be considered.^23^,^24^ Finally, depression is often associated with cognitive impairment, including deficits in attention, concentration, and executive function,^32^ which may influence performance on the General Psychopathology Scale, including items related to disorientation, poor attention, and motor retardation. For example, specific neurodegenerative processes, as evidenced with retina assessments,^33^,^34^ may influence schizophrenia symptom severity.

Second, our findings showed that people with depression have fewer positive symptoms than their non-depressed counterparts. Although this is not entirely consistent with other studies in this field,^8^,^35^ some relevant explanations can be hypothesized. In particular, it has been shown that depressive symptoms are more common in chronic schizophrenia, i.e., during “post-psychotic depression.”^36^,^37^ Since depressive symptoms may become apparent only as the positive symptoms recede,^12^ in our acutely ill sample, they might have been somehow obscured by the severity of hallucinations and delusions. However, several psychological models suggest that post-psychotic depression may encompass cognitive processes involving the restoration of insight.^37^ For instance, since insight is likely related to the emergence of depressive symptoms when the severity of psychotic features is reduced,^38^ it has been hypothesized it has a mediating or moderating effect on the relationship between depressive and positive symptoms. Alternative explanations involve a possible continuum between psychotic symptoms and affective features, ranging from depressed to elated mood, in acutely ill people with schizophrenia.^39^ A substantial portion of individuals presenting with predominantly positive symptomatology may be more likely to show mood elation, thus reporting lower CDSS scores. However, the occurrence of depressive symptoms in schizophrenia does not necessarily imply the presence of a comorbid major depressive disorder.^40^ Evidence seems to suggest some shared abnormalities in dopamine transmission, even though different areas of the central nervous system may be involved, namely the hippocampus and prefrontal cortex in schizophrenia and the medial frontal cortical regions and amygdala in depression.^40^

Finally, we could not find a clinical relationship between depressive and negative symptoms, whose differentiation remains a challenge due to their somehow inevitable overlap.^16^ While depressive symptoms might fluctuate more acutely, negative symptoms are likely to be correlated with chronic schizophrenia.^41^ Nevertheless, appropriate psychometric tools can assess different clinical dimensions in SSD since negative symptoms represent an independent cluster that can be clearly differentiated from depressive symptoms.^6^,^18^ Thus, it seems confirmed that the CDSS^42^ can be used to assess depression as a distinct domain, giving more weight to subjective experiences in terms of hopelessness, self-depreciation, and guilt than other assessment measures routinely used for depressive disorders, such as the Hamilton Depression Rating Scale.^43^

Several limitations should be considered when interpreting our findings. First, the cross-sectional design prevented us from establishing any causal relationship between depressive symptoms and the explored variables. Second, the participants were recruited from an inpatient setting, which may have resulted in overrepresentation of individuals with more severe symptoms. Third, the generalizability of our findings could also be limited in that some individuals not meeting the criteria for depression during the study period might have previously suffered from depression or were benefiting from antidepressant/mood stabilizing treatments. Furthermore, we should acknowledge some potential measurement issues, since the Brief Negative Symptom Scale could be used to assess negative symptoms alone,^44^ and the alternative five-factor PANSS model may perform better than the standard model.^45^ In addition, we did not test important variables that could influence both depressive symptoms and other clinical dimensions of schizophrenia, such as insight^46^ or the chlorpromazine equivalent daily dose of antipsychotic agents.^47^ Finally, we did not consider the possible confounding effects of antipsychotic-related extrapyramidal symptoms on both depressive and secondary negative symptoms.^6^

We could provide additional insight on the clinical relationship between depressive symptoms and key psychopathological features of schizophrenia. People with clinically significant depressive symptoms have more overall and general psychopathology symptoms, though lower levels of positive symptoms, while negative symptoms remain independent of depression. This is consistent with emerging approaches focusing on the characterization of salient domains and clinical dimensions of schizophrenia that, along with positive and negative symptoms, may include other psychopathological components, such as depression.^48^,^49^ Additional studies are needed to confirm the generalizability of our findings and to better delineate the clinical boundaries of depressive symptoms in schizophrenia.

## Disclosure

FB has received consultant fees from Edra SpA and IQVIA Solutions Italy, and honoraria for editorial activities from Elsevier, IMR Press, and Med Reviews Ltd. The other authors report no conflicts of interest.

## Figures and Tables

**Table 1:** Sample characteristics and differences between individuals with and without depression

Variables	Overall sample (n=231)	With depression (n=78)	Without depression (n=153)	Test statistic	p-value
Sociodemographic characteristics					
Age (years) (mean±SD)	42.4±12.9	42.7±13.1	42.2±12.9	-0.299 (z)	ns
Male sex	136 (58.9)	45 (57.7)	91 (59.5)	0.068 (χ^2^)	ns
Married/in a relationship†	39 (17.1)	15 (19.5)	24 (15.9)	0.463 (χ^2^)	ns
Education (years) (mean±SD)‡	10.9±3.3	10.7±3.1	10.9±3.4	0.384 (z)	ns
Unemployed§	156 (68.1)	53 (68.8)	103 (67.8)	0.027 (χ^2^)	ns
Clinical characteristics					
Compulsory treatment	82 (35.5)	23 (29.5)	59 (38.6)	1.858 (χ^2^)	ns
Suicide attempts||	23 (10.0)	13 (16.7)	10 (6.6)	5.743 (χ^2^)	0.017
Alcohol use disorder	27 (11.7)	14 (18.0)	13 (8.5)	4.471 (χ^2^)	0.034
Substance use disorder	66 (28.6)	21 (26.9)	45 (29.4)	0.157 (χ^2^)	ns
Personality disorder	66 (28.6)	17 (21.8)	49 (32.0)	2.650 (χ^2^)	ns
PANSS (mean±SD)					
Overall	82.5±20.1	89.1±20.1	79.2±19.3	-3.661 (t)	< 0.001
Positive	21.6±8.4	20.8±8.1	22.1±8.5	1.348 (z)	ns
Negative	19.1±9.0	21.1±8.7	18.1±9.0	-2.730 (z)	0.006
General psychopathology	41.8±11.3	47.2±11.7	39.0±10.0	-5.267 (z)	< 0.001
Physical comorbidities					
Hypertension¶	33 (14.4)	14 (18.4)	19 (12.4)	1.483 (χ^2^)	ns
Obesity	34 (14.7)	12 (15.4)	22 (14.4)	0.042 (χ^2^)	ns
Dyslipidemia	25 (10.8)	11 (14.1)	14 (9.2)	1.313 (χ^2^)	ns
Diabetes††	22 (9.6)	9 (11.8)	13 (8.5)	0.654 (χ^2^)	ns
Metabolic syndrome‡‡	62 (27.2)	22 (29.3)	40 (26.1)	0.259 (χ^2^)	ns
Dysthyroidism	18 (7.8)	7 (9.0)	11 (7.2)	0.229 (χ^2^)	ns
Psychopharmacological treatment					
Typical antipsychotics§§	62 (27.1)	26 (33.8)	36 (23.7)	2.631 (χ^2^)	ns
Atypical antipsychotics||||	86 (37.4)	35 (44.9)	51 (33.6)	2.821 (χ^2^)	ns
Mood stabilizers¶¶	40 (17.4)	10 (12.8)	30 (19.7)	1.716 (χ^2^)	ns
Antidepressants†††	30 (13.0)	19 (24.4)	11 (7.2)	13.324 (χ^2^)	< 0.001
None‡‡‡	90 (39.3)	26 (33.8)	64 (42.1)	1.568 (χ^2^)	ns

Data presented as n (%), unless otherwise specified.

ns = not significant (p > 0.05); PANSS = Positive and Negative Syndrome Scale.

Missing data: ^†^ n=3 (one with depression, two without depression); ^‡^ n=25 (seven with depression, 18 without depression); ^§^ n=2 (one with depression, one without depression); ^||^ n=2 (two without depression); ^¶^ n=2 (two with depression); ^††^ n=2 (two with depression); ^‡‡^ n=3 (three with depression); ^§§^ n=2 (one with depression, one without depression); ^||||^ n=1 (one without depression); ^¶¶^ n=1 (one without depression); ^†††^ n=1 (one without depression); ^‡‡‡^ n=2 (one with depression, one without depression).

**Table 2:** Association between depression and PANSS scores: multiple regression analyses

PANSS	Odds ratio† (95%CI)	Coefficient (SE)	p-value
Overall‡	1.03 (1.01-1.05)	0.029 (0.008)	< 0.001
Positive§	0.92 (0.87-0.97)	-0.088 (0.028)	0.002
Negative§	0.97 (0.93-1.02)	-0.026 (0.021)	0.210
General psychopathology§	1.13 (1.08-1.18)	0.118 (0.023)	< 0.001

PANSS = Positive and Negative Symptoms Scale.

† For each 1-unit increase.

‡ Model 1: adjusted for age, sex, antidepressant treatment, history of suicide attempts, and alcohol use disorder.

§ Model 2: adjusted for age, sex, antidepressant treatment, history of suicide attempts, alcohol use disorder, and other PANSS subscores.
